# Supplementary material for: Terrestrial Animal Source Foods and Health Outcomes for Those with Special Nutrient Needs in the Life Course
Source: Nutrients. 2024 Sep 24;16(19):3231. doi: 10.3390/nu16193231 (PMC11478082; doi:10.3390/nu16193231)
Supplement: Supplementary file 1 [file nutrients-16-03231-s001.zip › nutrients-3161525-supplementary.pdf]

Supplementary Materials

Table S1. Search terms used in the review by database..1

Table S1. Search terms used in the review by database

Search terms using Academic Search Complete via EBSCOHost
("animal source food" OR "animal sourced food" OR "animal based food" OR "livestock derived foods" OR "animal derived foods" OR "TASF" OR "meat" OR "milk" OR eggs) AND ("child" OR "Infant" OR "adolescent" OR "adult" OR "elderly" OR "school-age child" OR "child under the age of 5" OR "schoolchildren" OR "older adults" OR "breastfeeding" OR "lactation" OR "teenager") AND ("health" OR "nutrition)
**Search terms using PubMed**
("animal source food" OR animal source food* OR "animal based food" OR "livestock derived foods" OR "TASF" OR "meat" OR "milk" OR "eggs" OR "dairy" OR "insects" OR "honey") AND ("child" OR child* OR "infant" OR infant* OR "adolescent" OR "adult" OR "elderly" OR "school-age child"* OR "child under the age of 5" OR "older adults" OR "breastfeeding" OR "lactation" OR "teenager") AND ("health" OR "nutrition") AND ("meta-analysis" OR "systematic review" OR "experimental trial" OR "observational study")
**Search terms using ScienceDirect**
("animal source food" OR "animal sourced food" OR "animal based food" OR "livestock derived foods" OR "TASF" OR "meat" OR "milk" OR "eggs" OR "dairy" OR "insects" OR "honey") AND ("child" OR "Infant" OR "adolescent" OR "adult" OR "elderly" OR "school-age child" OR "child under the age of 5" OR "older adults" OR "breastfeeding" OR "lactation" OR "teenager") AND ("health")
